# Surgical treatment of a massive bilateral pulmonary embolus due to an entrapped thrombus in a patent foramen ovale: a case report

**DOI:** 10.1186/s13256-015-0527-6

**Published:** 2015-03-04

**Authors:** Edvin Prifti, Fadil Ademaj, Arben Baboci, Albana Doko, Daniela Teferici

**Affiliations:** 1 Division of Cardiac Surgery, University Hospital Center of Tirana, Tirana, Albania; 2 Division of Heart Disease, Gjakova Hospital, Rr Prizren, Gjakove, Kosovo

**Keywords:** Embolism, Entrapped, Foramen ovale, Patent, Pulmonary, Thrombus

## Abstract

**Introduction:**

Entrapped thrombus in a patent foramen ovale is a rare form of right heart thromboembolism. Various treatments have been used, such as anti-coagulation and thrombolytic therapy, vena cava filter, percutaneous thrombectomy and surgical embolectomy.

**Case presentation:**

A 60-year-old Kosovan woman was admitted to our hospital with a massive bilateral pulmonary thromboembolism, entrapped thrombus in the patent foramen ovale and severe right ventricular dysfunction. The patient underwent on-pump beating-heart removal of the intracardiac thrombus and bilateral pulmonary embolectomy with the use of a Fogarty catheter. The patient’s post-operative course was uneventful. In this report, we describe for the first time in this pathology, to the best of our knowledge, a surgical strategy that seems to offer an excellent outcome in patients with severe right ventricular dysfunction.

**Conclusions:**

The chosen surgical technique, consisting of on-pump open beating-heart surgery, is a unique procedure in the treatment of an acute pulmonary thromboembolism and entrapped thrombus in a patent foramen ovale.

## Introduction

The presence of a patent foramen ovale (PFO) in acute pulmonary embolism is associated with a significantly higher incidence of death and embolic complications [1]. A thrombus entrapped in a PFO is a rare form of right heart thromboembolism. In-hospital mortality of thrombus in transit is estimated to exceed 45% [2]. The first documented case of impending paradoxical embolism or entrapped thrombus through the PFO was reported in 1985 [3]. Various treatments have been described, such as anti-coagulation and thrombolytic therapy, vena cava filter, percutaneous thrombectomy and surgical embolectomy [2,4,5]. In this report, we describe a case of a patient with an entrapped thrombus through a PFO associated with a massive bilateral pulmonary embolism who underwent successful emergent surgical treatment, representing the first reported case in an emerging country, to the best of our knowledge. The surgical technique chosen, consisting of on-pump open beating-heart surgery, is a unique procedure in the treatment of an acute pulmonary embolism and entrapped thrombus in a PFO.

## Case presentation

**Figure 1:**
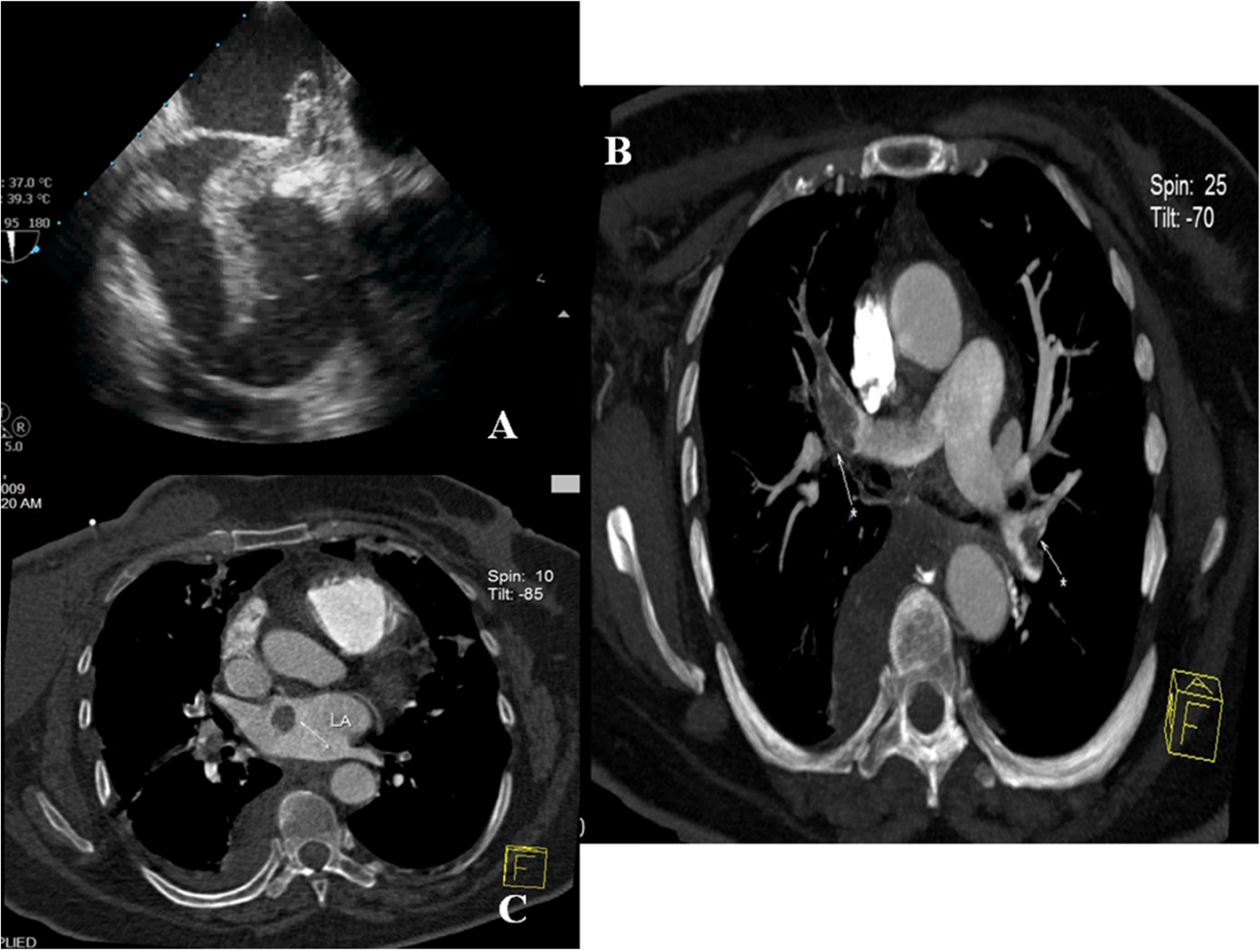
**Pre-operative imaging studies. (A)** Pre-operative transthoracic echocardiogram shows an entrapped thrombus in the foramen ovale. **(B)** Pre-operative contrast-enhanced computed tomographic scan shows a massive bilateral thromboembolism (white arrows). **(C)** Pre-operative contrast-enhanced computed tomographic scan shows the intracardiac thrombus (white arrow).

A 60-year-old Kosovan woman was admitted at the Division of Cardiac Surgery of our hospital on an emergency basis with an acute onset of exertional dyspnea associated with chest pain. Her medical history was positive for hypertension and hypercholesterolemia. A month before, she had undergone a left-side total mastectomy for treatment of breast cancer. She presented with sinus rhythm, tachycardia, tachypnea, and pulse oximetry of 72% on room air. Her cardiac enzymes were normal. An urgent transthoracic echocardiography was performed, which demonstrated an embolus extending from the right atrium to the left atrium through the PFO (Figure 1A). Severe right ventricular dysfunction was present, with a fractional area grade of almost 14%. Pulmonary hypertension of 75mmHg was measured, which was associated with poor right ventricular function. Immediately, contrast-enhanced computed tomography (CT) was performed, which revealed severe bilateral pulmonary artery emboli (Figure 1B) and a right atrial mass (Figure 1C). Coronary angiography demonstrated a normal coronary tree.

The patient underwent an emergent on-pump beating-heart surgical pulmonary and bilateral pulmonary embolectomy. A bicaval and standard aortic cannulation was performed. An extra 6-French line was extended from the aortic line to the cardioplegic cannula. After cardiopulmonary bypass was initiated, the ascending aorta was clamped, and the line connected to the aortic line with a cardioplegic cannula was unclamped, making possible delivery of almost 800ml of blood per minute to the coronary arteries. The patient’s heart was maintained in sinus rhythm during the operation. The right atrium was opened, and the migrating thrombus across the PFO was pulled out. The interatrial septum was opened widely, and the left atrium was inspected carefully and then closed after de-airing. The right atrium was closed with a direct 4–0 continuous PROLENE™ polypropylene suture (Ethicon, Somerville, NJ, USA). Next, the main pulmonary artery was opened and extended toward the left pulmonary artery, and a 5-French Fogarty catheter was employed to remove the thrombus from the left pulmonary artery and its branches. The same procedure was attempted to the right pulmonary artery through the same incision, but it was unsuccessful. Next, the right pulmonary artery close to the superior vena cava was opened. The superior venous cannula was removed and repositioned on the superior caval vein, which was then detached from the right atrium. The incision on the right pulmonary artery was extended toward the right pulmonary hilus, which was opened completely, and the thrombus was removed. The embolectomy of the branches of the right pulmonary artery was performed using the same Fogarty catheter. Next, the hilus was reconstructed using an autologous pericardial patch, and the right pulmonary artery was closed. The superior vena cava was then anastomosed to its usual position at the right atrium. The air was removed carefully from the heart, and the aorta was declamped.

**Figure 2:**
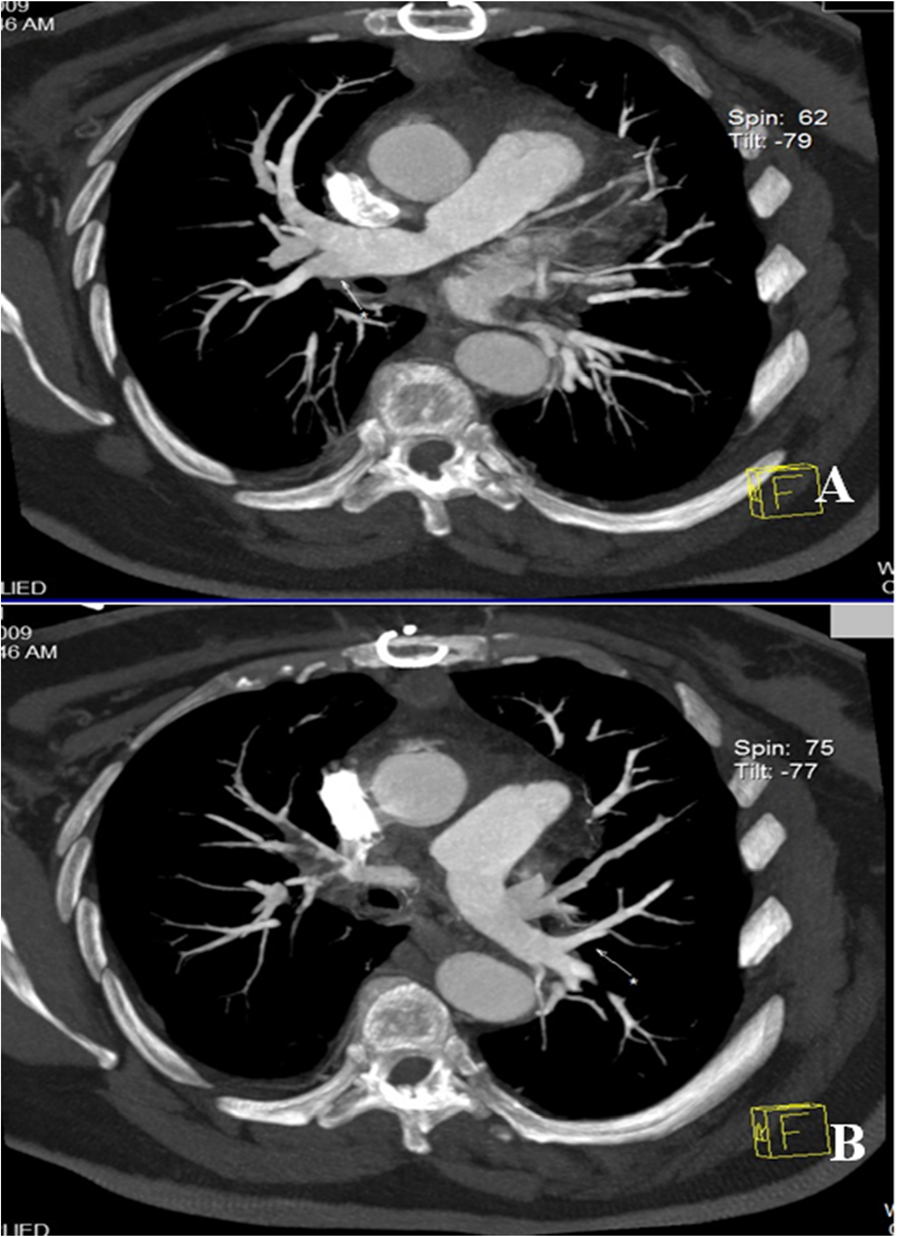
**Post-operative imaging studies. (A)** Post-operative contrast-enhanced computed tomographic scan shows total removal of the thrombus on the right pulmonary artery. **(B)** Post-operative contrast-enhanced computed tomographic scan shows total removal of the thrombus on the left pulmonary artery.

The patient’s post-operative course was uneventful, and she was discharged on the eighth post-operative day. Post-operatively, the patient received daily warfarin treatment for 1 year to maintain a 2.5 to 3.5 international normalized ratio. At 1 month after surgery, contrast-enhanced CT showed a normal pulmonary tree (Figure 2A,B). At 1 month, the patient underwent inferior caval filter implantation at another institution in a European Community country. At 1 year after surgery, the patient was still alive and free of symptoms.

## Discussion

One-third of patients with deep venous thrombosis can present with pulmonary embolisms and have a mortality of approximatively 12% [6]. Floating right heart thrombi are uncommon, and they are always associated with acute pulmonary embolisms. Paradoxical movement of the thrombus across a PFO indicates reversal of flow from the right atrium to the left atrium due to acute pulmonary embolism. Floating thrombi entrapped in a PFO are extremely rare. In a careful review of the literature, we found almost 111 reported cases. Pulmonary embolism was most frequently found, accounting for almost 60% of the cases. Conversely, isolated systemic embolus is rare. Only three cases are known [6], even though echocardiography is generally performed after any stroke or distal acute ischemia, which would be able to detect such an embolus. Almost 37% of cases presented with pulmonary and systemic emboli [7]. The reason for this could be that a high right atrial pressure is necessary to protrude a thrombus through the inter-atrial shunt [5,8].

In most cases, the diagnosis is made correctly on the basis of transthoracic echocardiography, and, when there is doubt, transesophageal echocardiography can be employed successfully [9]. To the best of our knowledge, there is no report of a false-positive diagnosis in the literature. Surprisingly, more than one-half of the reported cases had an atypical clinical presentation without a paradoxical or pulmonary embolism. This could be an additional reason to perform routine echocardiography after any pulmonary or arterial embolism. Echocardiography is the first-line diagnostic tool for ruling out valvular heart disease and diagnosing the presence of an entrapped thrombus. This modality can help to determine right heart tension, pulmonary pressure and cardiac function in cases of pulmonary emboli, which are important markers for choosing the right treatment option.

In our patient, we detected an entrapped thrombus at the PFO, most probably due to deep venous thrombosis, but without systemic embolization. A Doppler examination revealed thrombosis of the deep veins of the right leg. In such cases, duplex scanning of abdominal and deep veins of the legs is also recommended, especially to examine the possibility of placing an inferior caval filter or clip. It is prudent to perform pulmonary CT angiography as a first-line approach to assess for the presence of pulmonary embolism.

The best management is still controversial. Three therapeutic options can be discussed. The impending thrombus can be removed surgically, or it can be dissolved by thrombolytics and/or anti-coagulation. Studies have found that almost 65% of such patients underwent surgery, and the mortality rate was 9.7% versus 36% and 32% mortality in patients who underwent thrombolysis [10] or anti-coagulation therapy [11]. Theoretically, thrombolysis and even anti-coagulation therapy seem to be hazardous in large, mobile intracardiac thrombi, with an important risk of either fragmentation or complete embolization. Moreover, deep vein thrombosis can be a source of recurrent embolisms. Conversely, the delay between clinical presentation and echographic diagnosis can be extremely variable, from 1 hour to a few weeks. In the cases with long delays, the efficacy of thrombolytics or anti-coagulants might be more hypothetical on an old thrombus.

We preferred surgery in our patient, even though we were aware of the risks of cardiac surgery, as the patient had cardiac failure. This therapeutic choice seemed to be more rapid and complete than the others, as the PFO would be closed at the same time [12]. No embolization after surgical thrombus removal is reported. In our patient, we modified the usually employed technique. Because of our patient’s severe right ventricular dysfunction, we preferred to perform on-pump open beating-heart surgery, which permitted us to remove the entrapped thrombus in the PFO and all of the thrombus extending into the pulmonary artery. Such a technique has been reported in the treatment of ischemic heart disease [13] and mitral valve disease [14] in patients with low left ventricular ejection fraction. We believe that the surgical strategy we employed is an optimal procedure, especially in cases like our patient’s with a presentation of severe right ventricular dysfunction.

## Conclusions

On the basis of our experience and our review of the reported cases of entrapped thrombus in a PFO associated with pulmonary thromboembolism, we conclude that the surgery we performed seems to have the best results among the therapeutic options for these patients. In addition, anti-coagulation or thrombolysis can pose secondary complications, especially recurrent embolisms.

## Consent

Written informed consent was obtained from the patient for publication of this case report and any accompanying images. A copy of the written consent is available for review by the Editor-in-Chief of this journal.

## Abbreviations

CT: Computed tomography
PFO: Patent foramen ovale
